# Subcutaneous Division of the Thigh-Bone

**Published:** 1870-09

**Authors:** 


### Subcutaneous Division of the Thigh-Bone.

Subcutaneous surgical practice has made a remarkable advance during the present month. In the latter part of November a man was admitted into the Great Northern Hospital under the care of Mr. Wm. Adams, with anchylosis of the hip-joint, the result of a rheumatic fever suffered seven years ago. The limb of the patient being so deformed as to be utterly useless, Mr. Adams determined to make a subcutaneous division of the neck of the thigh bone, within the capsular ligament. He performed the operation on the 1st of December, piercing to the bone with a long small knife, and dividing the bone itself with a fine saw. The leg was brought, immediately after the division of the neck of the bone, into a straight position, and fixed into a long splint, and the case has progressed with not one bad sympton, and the wound has closed without any inflammatory action or suppuration. The splint has been removed, and the man can move the thigh to a limited extent. Whether motion of the limb can be preserved remains to be proved, and, if it cannot, the limb will be transferred from a useless to a useful condition; but the great value of the case is that it establishes as a fact the possibility of performing so important an operation subcutaneously, and without an untoward symptom as a result. The operation will be a mark, in the year now nearly over, of the triumph of subcutaneous surgery.—*Lancet.*

